# Osmotic stress in banana is relieved by exogenous nitric oxide

**DOI:** 10.7717/peerj.10879

**Published:** 2021-02-09

**Authors:** Muhammad Asyraf Mohd Amnan, Teen-Lee Pua, Su-Ee Lau, Boon Chin Tan, Hisateru Yamaguchi, Keisuke Hitachi, Kunihiro Tsuchida, Setsuko Komatsu

**Affiliations:** 1 Centre for Research in Biotechnology for Agriculture, University of Malaya, Kuala Lumpur, Malaysia; 2 Department of Medical Technology, Yokkaichi Nursing and Medical Care University, Yokkaichi, Japan; 3 Institute for Comprehensive Medical Science, Fujita Health University, Toyoake, Japan; 4 Faculty of Life and Environmental and Information Sciences, Fukui University of Technology, Fukui, Japan

**Keywords:** Banana, Drought, Nitric oxide, Osmotic stress, Proteomics, Reactive oxygen species

## Abstract

Drought is one of the severe environmental stresses threatening agriculture around the globe. Nitric oxide plays diverse roles in plant growth and defensive responses. Despite a few studies supporting the role of nitric oxide in plants under drought responses, little is known about its pivotal molecular amendment in the regulation of stress signaling. In this study, a label-free nano-liquid chromatography-mass spectrometry approach was used to determine the effects of sodium nitroprusside (SNP) on polyethylene glycol (PEG)-induced osmotic stress in banana roots. Plant treatment with SNP improved plant growth and reduced the percentage of yellow leaves. A total of 30 and 90 proteins were differentially identified in PEG+SNP against PEG and PEG+SNP against the control, respectively. The majority of proteins differing between them were related to carbohydrate and energy metabolisms. Antioxidant enzyme activities, such as superoxide dismutase and ascorbate peroxidase, decreased in SNP-treated banana roots compared to PEG-treated banana. These results suggest that the nitric oxide-induced osmotic stress tolerance could be associated with improved carbohydrate and energy metabolism capability in higher plants.

## Introduction

Water deficit caused by soil drought is one of the main threats affecting banana growth and production (Muthusamy et al., 2016). It disrupts the cellular redox homeostasis, which leads to oxidative stress and causes injury and cell death (Zhou et al., 2020). To survive, plants respond and adapt to drought stress by changes at morphological, physiological, biochemical, and molecular levels (Hai et al., 2020). At the morphological level, plant size, leaf expansion, and biomass decreased under drought conditions (Khamis et al., 2019). In banana, water stress severely reduced the emergence of new leaves, leaf area, and plant height (Mahouachi, 2009). At the physiological and biochemical levels, drought-induced stomatal closure, osmolyte accumulation, and reactive oxygen species (ROS) scavenging mechanism; and synthesized protective proteins, such as dehydrins, heat shock proteins, and late embryogenesis abundant proteins, are strategies for plants to cope with drought stress (Li et al., 2019). At the molecular level, a set of genes and signal transduction pathways have been discovered to play a significant role in drought stress responses (Haider et al., 2018). These include abscisic acid (ABA), strigolactone, lipid-derived signal, ROS, and soluble sugars, which were mediated by ABA-independent and ABA-dependent regulatory pathways (Du et al., 2018). These findings suggest that different strategies might be used by banana plants to adapt and defend themselves from drought condition.

Few strategies have been adopted to alleviate plants from drought stress, including developing drought-tolerant plants via conventional breeding, genetic engineering, introducing exogenous hormones, such as salicylic acid, or exogenous signaling molecules, such as nitric oxide. Conventional breeding, such as wide-cross hybridization and mutation breeding, and modern biotechnology, such as genetic engineering, were used to produce crop cultivars with various stress-tolerant traits (Anwar & Kim, 2020). Exogenous application of signaling molecules, such as salicylic acid and nitric oxide, is a well-known stress mitigation strategy (Chavoushi et al., 2019). Nitric oxide is a water-lipid-soluble free radical gaseous and redox related signaling molecule, which was rapidly produced by multiple hormonal and environmental stimuli. Nitric oxide played an important role in cytoprotection by regulating the level of ROS and by inducing transcriptional changes of a wide range of targets leading to the modulation of protein function (Zhao et al., 2020). As a signaling molecule, nitric oxide is involved in several plant-growth processes, such as germination, root/leaf development, respiration, and photosynthesis (De Sousa et al., 2020). It plays roles in defense-related biological processes, such as abiotic and biotic stresses (Munawar et al., 2019). These findings underline the importance of nitric oxide in plant growth and development. However, the mechanism of nitric oxide-mediated signaling in plants remains unknown.

The involvement of nitric oxide in alleviating drought stress first reported in wheat seedlings (García-Mata & Lamattina, 2001). Exogenous supply of nitric oxide enhanced plant tolerance to drought stress by reducing water stress/ion leakage/transpiration rate, increasing antioxidant activity, accelerating protein synthesis, enhancing photosynthetic capacity, and maintaining tissue-water potential (Fan et al., 2012). Since direct exposure to nitric oxide is technically difficult, these studies mostly rely on exogenous application of nitric oxide donors, such as sodium nitroprusside (SNP), S-nitro-N-acetylpenicillamine, and S-nitrosoglutathione. Of these, SNP is a widely used nitric oxide donor (Chavoushi et al., 2019). The efficiency of the nitric oxide release of the donors S-nitrosoglutathione, nitric oxide synthase, and SNP is different. A previous study showed that S-nitrosoglutathione is not an efficient nitric oxide generator as it failed to induce accumulation of the target gene, *AOX1a*, transcripts and hydrogen peroxide (H_2_O_2_) due to an inefficiency of nitric oxide generated (Ederli et al., 2009). The beneficial effects of nitric oxide depend on various factors, such as concentration, treatment duration, and application methods of nitric oxide donors. Exposure of relatively high doses of nitric oxide influenced normal metabolism and reduced respiration, which may lead to cytotoxicity (Santisree, Bhatnagar-Mathur & Sharma, 2015). Low concentrations of SNP application attenuated the negative effects of water deficit in *Physalis angulata* and improved photosynthetic rates and growth (Da Silva Leite et al., 2019). Despite the importance of nitric oxide in plant tolerance to abiotic stresses, the details about the nitric oxide-modulated response on proteins are still unclear and need further exploration.

In banana, proteins related to the stress/defense, energy metabolism, and heat shock proteins accumulated under stress condition (Ji et al., 2019). Analysis of protein profiles in gels of five banana varieties subjected to mild osmotic stress showed that respiration, ROS metabolism, and several dehydrogenases involved in NAD/NADH homeostasis were changed (Vanhove et al., 2012). These studies mainly focused on identifying proteins involved in banana response under drought condition. Although few studies have examined the effect of nitric oxide in plant growth (Nabi et al., 2019), the role and mechanism of nitric oxide on drought stress amelioration in banana have not been determined. In this study, polyethylene glycol (PEG) was used to simulate a drought-like stress condition. Morphological analysis of osmotic-stressed and nitric oxide-treated bananas at different periods was performed. Based on the morphological results, well-watered, PEG-treated, and PEG+SNP-treated bananas were selected for analyzing antioxidant enzyme activities and proteomic analysis.

## Materials & Methods

### Overview of current study

**Figure 1:**
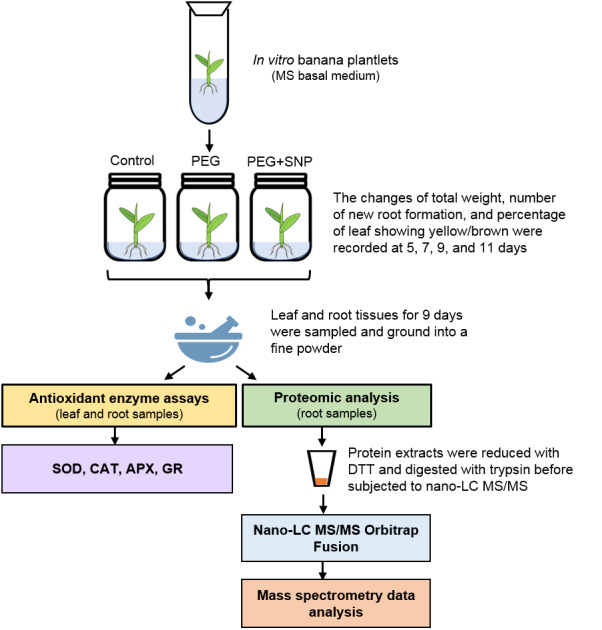
The schematic diagram of the experimental setup.

The influence of nitric oxide on osmotic stress amelioration was investigated using in vitro banana plantlets. PEG was used to mimic osmotic stress because of its effectiveness in decreasing medium water potential and incapability of penetrating plant cell wall due to its high molecular weight. The in vitro banana plantlets cultured in liquid medium containing PEG with or without SNP were evaluated for their morphological changes at 5, 7, 9, and 11 days. Total proteins from untreated root (control), root treated with PEG alone (PEG) or PEG and SNP (PEG+SNP) for 9 days were extracted for proteomics analysis. Activities of superoxide dismutase (SOD), catalase (CAT), ascorbate peroxidase (APX), and glutathione reductase (GR) in leaf and root samples for each treatment were determined at day 9. The schematic diagram of the experimental setup is presented in Fig. 1.

### Plant material and treatment

In vitro banana plantlets (*Musa acuminata* cv. Berangan) purchased from the Felda Global Holding (Bandar Baru Enstek, Malaysia) were maintained in liquid Murashige and Skoog medium (Murashige & Skoog, 1962) containing 5% (w/v) PEG with or without 5 µM SNP. SNP (5 µM) was used based on our preliminary experimental results (data not shown). An aqueous solution of 1.0 mM SNP was prepared fresh and wrapped in aluminum foil to prevent light-induced degradation. The liquid Murashige and Skoog medium without both 5% (w/v) PEG and 5 µM SNP supplement served as the control because it is the commonly used medium for banana tissue culture plantlets. The pH of the Murashige and Skoog media was adjusted to 5.8 prior to autoclaving. The total dry weight of plants after treatment and before treatment was recorded at 5, 7, 9, and 11 days. The number of root formation was determined by counting the number of new roots (>0.5 cm length) formed at 9 and 11 days. Leaf yellowing was determined by categorizing the leaves into three groups according to their color (green, green/yellow, and yellow/brown). The percentage of leaf showing yellow/brown was recorded at 11 days. The experiments were conducted with a total of 3 plantlets per treatment and were repeated thrice. All cultures were incubated at 25 °C under a 16-h light, and 8-h dark cycle.

### Relative water content

Relative water content (RWC) was determined as described by Turner (1981). Fresh weight (FW) of leaves was measured immediately after harvest. The leaf samples were then submerged in water for 6 h until fully turgid. The turgid weight (TW) of the samples was measured. Next, the leaves were oven dried for 2 days and reweighed to obtain the dry weight (DW). The RWC was calculated using the following formula: RWC (%) = (FW − DW) / (TW − DW) × 100.

### Protein extraction, enrichment, reduction, alkylation, and digestion

A portion (300 mg) of roots was cut into small pieces and transferred to a filter cartridge. It was ground with a plastic rod for 60 times before adding 50 µL of cold detergent-free lysis buffer containing 7 M urea, 2 M thiourea, 5% 3-((3-cholamidopropyl) dimethylammonio)-1-propanesulfonate, and 2 mM tributylphosphine to the filter cartridge. The sample was ground for another 30 times. Lysis buffer (50 µL) was added to the sample and ground for 30 times. The resulting suspension was centrifuged at 15,000 ×*g* for 2 min and the supernatant was collected as total proteins. Protein concentrations were determined using bovine serum albumin as a standard in accordance with Bradford method (Bradford, 1976).

The extracted proteins (100 µg) were cleaned up according to Komatsu et al. (2013). After adjusting the volume to 100 µL, the protein extracts were mixed with 400 µL methanol before adding 100 µL chloroform/300 µL water. After mixing and centrifugation at 20,000 × g for 10 min, the lower phase was added with 300 µL of methanol and centrifuged again at 20,000 × g for 10 min. The pellet was collected as a soluble fraction (Komatsu et al., 2013) and resuspended in 50 mM ammonium bicarbonate. Proteins were reduced with 50 mM dithiothreitol for 30 min at 56 °C and alkylated with 50 mM iodoacetamide for 30 min at 37 °C in the dark before digested with trypsin (Wako, Osaka, Japan) at a ratio of 1:100 (enzyme/protein) for 16 h at 37 °C. The tryptic peptides were desalted using a MonoSpin C18 Column (GL Sciences, Tokyo, Japan) and equilibrated in 1% trifluoroacetic acid before subjected to nano liquid chromatography (LC) mass spectrometry (MS)/MS analysis.

### Identification of proteins using nanoLC MS/MS

The peptides were loaded onto an LC system (EASY-nLC™ 1000; Thermo Fisher Scientific, San Jose, CA, USA) equipped with a trap column (Acclaim PepMap 100 C18 LC column, 3 µm, 75 µm ID × 20 mm; Thermo Fisher Scientific) equilibrated with 0.1% formic acid. Peptides were eluted from the trapping column at a flow rate of 300 nL/min with a linear acetonitrile gradient (0–35%) in 0.1% formic acid onto a column (EASY-Spray C18 LC column, 3 µm, 75 µm ID × 150 mm; Thermo Fisher Scientific) with a spray voltage of 2 kV (Ion Transfer Tube temperature: 275 °C). The peptide ions were detected using MS (Orbitrap Fusion ETD MS; Thermo Fisher Scientific, USA) in the data-dependent acquisition mode with the installed Xcalibur software (version 4.0; Thermo Fisher Scientific, USA). Full-scan mass spectra were acquired in the *m/z* range of 375–1,500 with a resolution of 120,000. The most intense precursor ions were selected for collision-induced fragmentation using a normalized collision energy of 35%. The dynamic exclusion duration was set to 60 s to prevent repetitive selection of peptides (Zhang et al., 2009).

### Mass spectrometry data analysis

The MS data were searched using MASCOT search software (Version 2.6.1, Matrix Science, London, UK) and SEQUEST HT search algorithms against the *Musa acuminata* (Banana) UniProtKB protein database (2018-12-04) using Proteome Discoverer 2.2 (Version 2.2.0.388; Thermo Fisher Scientific, USA) using the following workflow: spectrum files RC, spectrum selector, MASCOT, SEQUEST HT search nodes, percolator, ptmRS, and minor feature detector nodes. The parameters of search were set as follows: Methionine oxidation as a variable modification and cysteine carbamidomethylation as a fixed modification, MS and MS/MS tolerances of 10 ppm and 0.6 Da, respectively, and one missed trypsin cleavage. Target-decoy database searches were used to calculate the false discovery rate (FDR). For peptide identification, FDR was set at 1%.

### Differential analysis of proteins using MS data

Label-free quantification was carried out using Proteome Discoverer 2.2. PERSEUS (version 1.6.2.3) (Tyanova et al., 2016) to analyze the relative abundance of peptides and proteins between samples and the values were transferred into log_2_ scale. Three biological replicates of each sample were grouped. A minimum of three valid values was required in at least one group. The values of both proteins and peptides abundances were normalized. Missing values were imputed based on a normal distribution (width = 0.3, down-shift = 1.8). The statistical significance was analyzed using *t*-test. The principal component analysis was performed with Proteome Discoverer 2.2.

### Functional categorization

The sequences of the differentially altered proteins were searched against the AmiGO database (http://amigo.geneontology.org/amigo) using a Basic Local Alignment Search Tool query. A Perl program was used to extract the gene ontology (GO) annotations from the most homologous proteins. The GO annotation results were analyzed using Web Gene Ontology Annotation Plot (WEGO; http://wego.genomics.org.cn/cgi-bin/wego/index.pl) tool. The gene functional annotations and protein categorization were analyzed using MapMan bin codes (Usadel et al., 2005) and protein abundance ratio was assessed through MapMan software (Usadel et al., 2009). The MapMan software is generally linked with several external databases (http://mapman.gabipd.org). The identified proteins were mapped to metabolic pathways in Kyoto Encyclopedia of Genes and Genomes (KEGG) databases (Kanehisa & Goto, 2000) (http://www.genome.jp/kegg/).

### Antioxidant enzyme assays

The leaves and roots of bananas treated with PEG alone or PEG+SNP for 9 days were sampled for antioxidant enzyme assays. The leaf and root samples were washed three times with distilled water. A portion (1 g) of each sample was ground into a fine powder in the presence of liquid nitrogen and extracted in lysis buffer containing 50 mM phosphate buffer (pH 7), 2% polyvinylpyrrolidone, and 0.01% phenylmethylsulfonyl fluoride. The protein extract was centrifuged twice at 20,817× g at 4 °C for 5 min and the supernatant fractions were frozen at −20 °C until use. The protein content was determined by the Bradford method (Bradford, 1976). All enzyme assays were performed in triplicate.

The SOD activity was determined according to Beauchamp & Fridovich (1971). The reaction containing a mixture of supernatant (50 µL), 200 mM phosphate buffer (pH 7.8), 250 µM nitro blue tetrazolium, 10 µM riboflavin, and 10 µL tetramethylethylenediamine. The mixture was incubated at room temperature for 15 min before measured at 560 nm using a UV–VIS spectrophotometer (Shimadzu, Kyoto, Japan). The enzyme activity (U mg^−1^ protein) was calculated by monitoring the reaction mixture at 30 and 60 s.

The CAT activity was determined according to Aebi (1984) with minor modifications. The 2.5 mL of assay mixture contained 50 µL sample in 50 mM potassium phosphate buffer (pH 7) and 0.036% (w/v) H_2_O_2_. After incubated at room temperature for 10 min, 1 mL sulfuric acid, and 1.45 mL distilled water were added to the mixture. The enzyme activity was determined by measuring the absorbance at 240 nm using a spectrophotometer. The extinction coefficient of H_2_O_2_ (39.4 µM^−1^ cm^−1^) was used to calculate the enzyme activity that was shown in terms of millimoles of H_2_O_2_ per minute per gram fresh weight.

The APX activity was measured according to Nakano & Asada (1981) with minor modification. The protein extract (0.1 mL) was added into in the reaction (1 mL) containing 50 mM sodium phosphate buffer (pH 7.0), 5 mM ascorbate, 1 mM H_2_O_2_, and 1 mM ethylenediaminetetraacetic acid. H_2_O_2_ was added last to initiate the reaction, and the decrease in absorbance was recorded for 3 min. The APX activity was estimated by monitoring the ascorbate oxidation rate (ε = 2.8 mM^−1^ cm^−1^) at 290 nm for 1 min with 30 s intervals.

The GR assay was carried out according to Halliwell & Foyer (1978). Adsorption of light by the co-substrate of GR (NADPH) was measured at wavelength 340 nm in reducing trends. The reaction mixture of 3 mL contained 0.1 mM NADPH, 1 mM oxidized glutathione (GSSG) and 100 mM potassium phosphate, pH 7.4. After adding enzyme extract, the reaction was measured for 2 min with 30 s intervals. The GR was calculated using a molar extinction coefficient of 6220 M^−1^ cm^−1^.

### Statistical analysis

Morphological data were recorded in control and treated banana plantlets. Each treatment consisted of 3 plantlets and the whole experiment was repeated thrice. In all experiments measuring activities of SOD, CAT, APX, and GR, each treatment contained three independent biological replicates and three technical replicates of each biological replicate. All data were analyzed statistically by one-way analysis of variance (ANOVA) followed by Duncan multiple comparisons among multiple groups at a significant level of *p* < 0.05 using SPSS (version 22.0; IBM, USA).

## Results

### Morphological alterations of PEG+SNP-treated bananas

**Figure 2:**
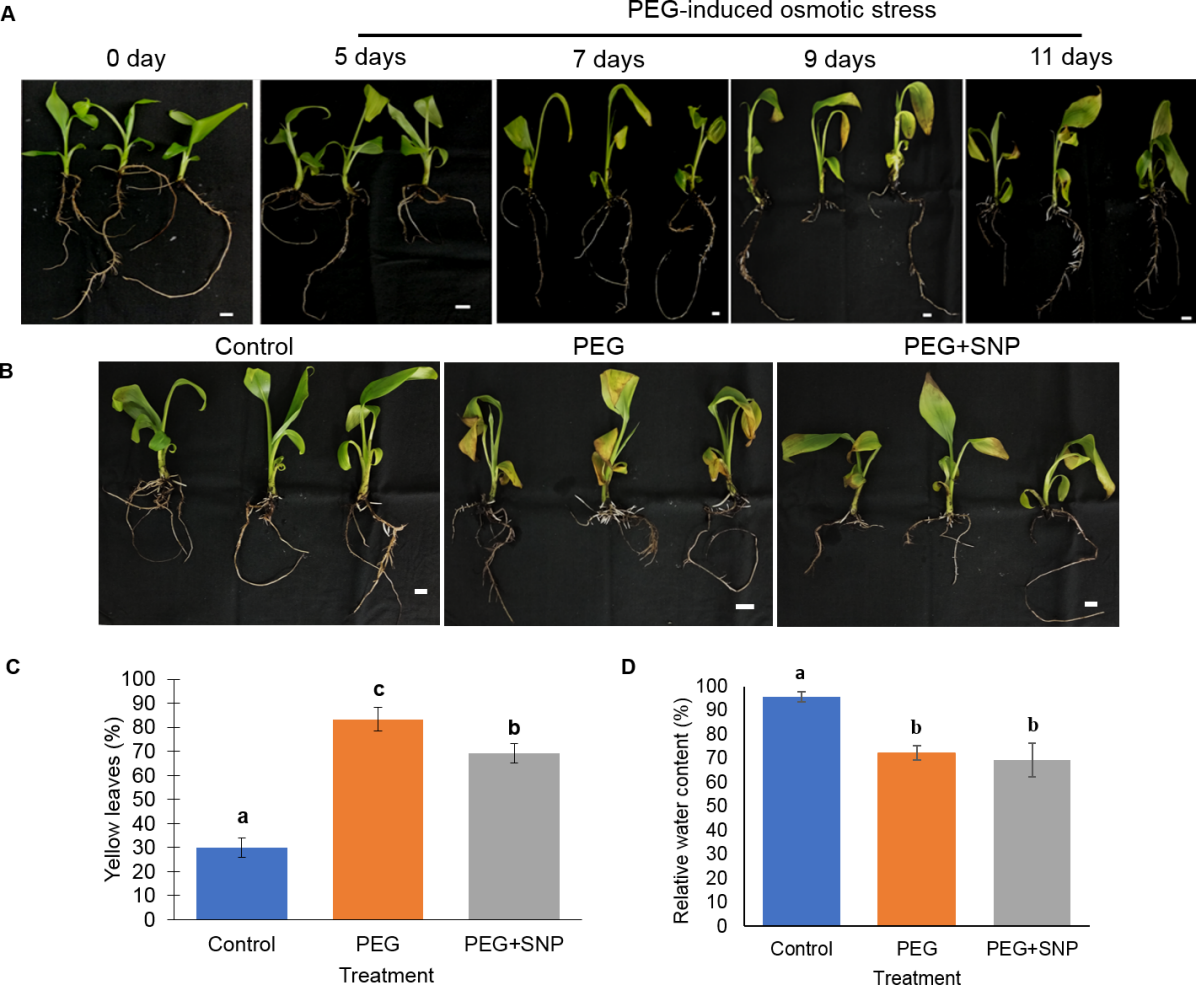
Influence of PEG-induced osmotic stress on banana. Banana plants were grown in culture media alone or containing PEG for 5, 7, 9, and 11 days. (A) Photographs of banana plants were taken at 0, 5, 7, 9, and 11 days. (B) Visible leaf yellowing symptoms, (C) the percentage of yellow leaves, and (D) the percentage of relative water content were recorded in banana plants grown in culture media containing PEG with or without SNP after 11 days of treatment. Leaf yellowing was observed daily until the leaves showed complete yellowing after 11 days of treatment. Culture media without PEG and SNP supplement was served as control. Data are shown as the means ± SE from three independent biological replicates. Means indicated with the different letters were significantly different based on analysis of variance (ANOVA) followed by Duncan’s multiple range test at *p* < 0.05. The scale bar indicates 1 cm.

**Figure 3:**
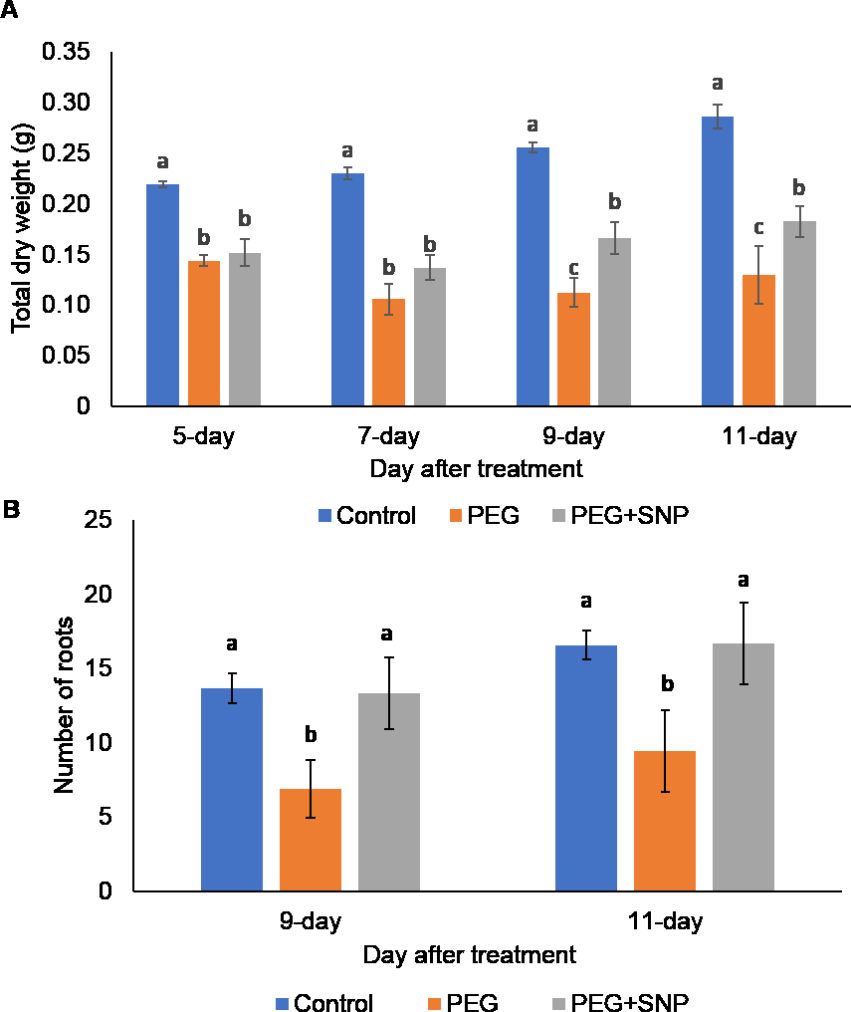
The changes of total weight and number of newly formed roots of banana plants treated with PEG with or without SNP. (A) The total weight of the plants was measured at 5, 7, 9, and 11 days of treatment, whereas (B) the number of newly formed roots was recorded at 9 and 11 days of treatment. Total weight data were log transformed prior to statistical analysis. Data are shown as the means ± SE from three independent biological replicates. Means indicated with the same letter were not significantly different based on analysis of variance (ANOVA) followed by Duncan’s multiple range test at *p* < 0.05.

The effects of PEG-treated osmotic stress without or with SNP on the growth of banana, such as the changes in percentage of yellow leaves (Fig. S1), RWC, total dry weight, and the number of new root formation, were evaluated. The leaves of bananas showed mild yellowing after 9 days of treatment (Fig. 2A). There was 64 out of 80 of the leaves (80%) showed yellowing in PEG-treated bananas after 11 days of treatment (Figs. 2B and 2C). The application of SNP together with PEG significantly reduced the percentage of yellow leaves (Figs. 2B and 2C). When compared with the control, a significant decrease in RWC was observed in the PEG-treated and PEG+SNP-treated bananas (Fig. 2D). The total dry weight of the PEG-treated and PEG+SNP-treated bananas was significantly reduced compared to control after 5 days of osmotic treatment (Fig. 3A). On the other hand, the total dry weight of bananas treated with PEG+SNP was significantly higher than that of PEG-treated bananas at 9 and 11 days. The number of newly formed roots was measured at 9 and 11 days after treatment as there were no visible new roots at days 5 and 7. The number of roots formed in the PEG+SNP-treated bananas was 13.3 and 16.7 after 9 and 11 days of treatment, respectively, which were comparable with well-watered bananas (13.7 and 16.6 roots after 9 and 11 days of treatment, respectively) (Fig. 3B). The number of new roots of banana treated with PEG was significantly lower than that of control and banana treated with PEG+SNP for both 9 and 11 days. Based on the morphological results, the 9-day root samples of each treatment were selected for proteomic analysis to determine the underlying molecular mechanisms of SNP on banana in response to osmotic stress.

### Changes of proteins in PEG+SNP-treated and osmotic-stressed bananas

**Figure 4:**
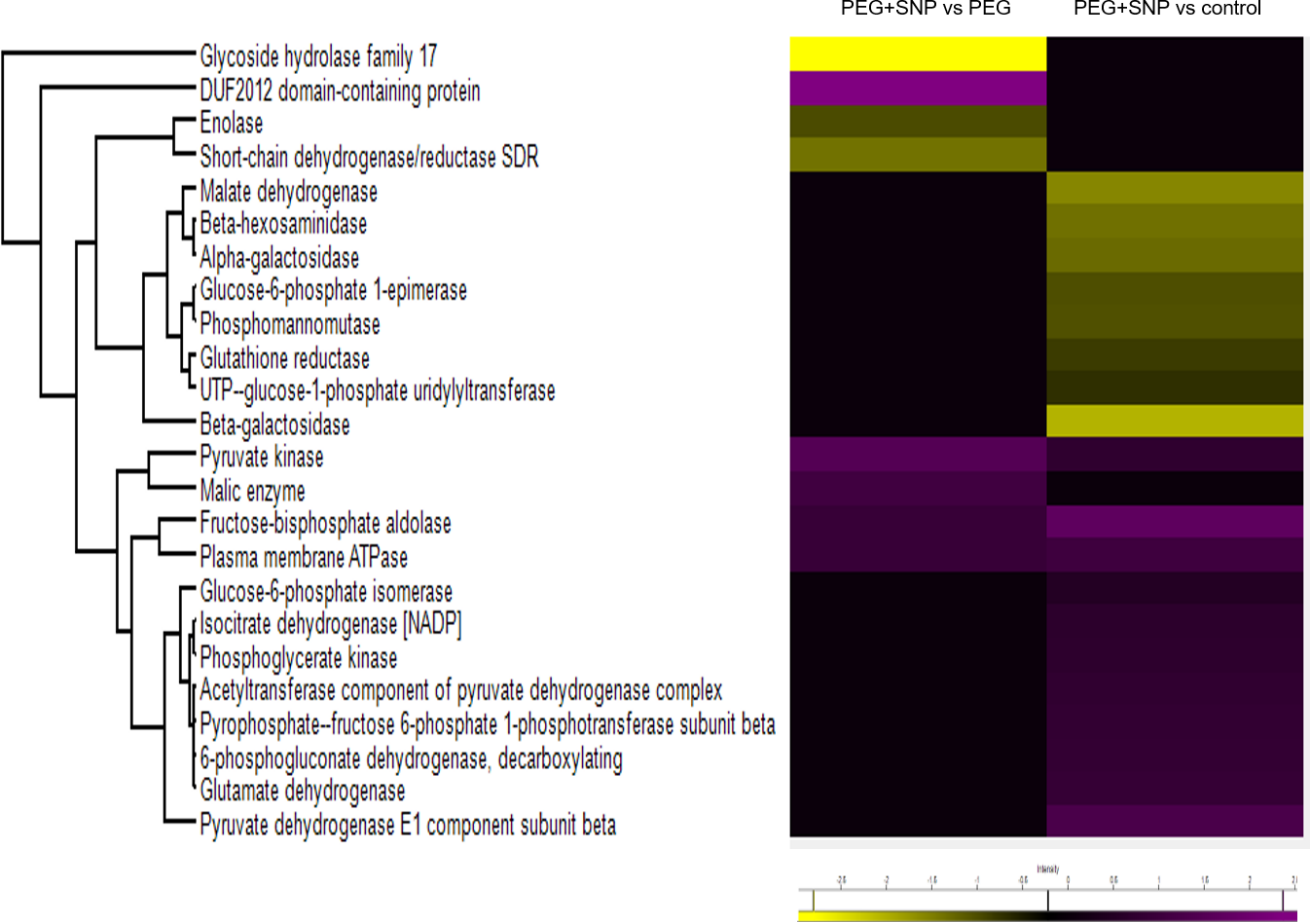
Heat map of the abundant carbohydrate metabolism-related proteins induced by osmotic stress and SNP in banana. Banana plants were exposed to PEG with or without SNP for 9 days. Proteins were extracted from the roots and analyzed using nanoLC-MS/MS. Violet and yellow colors indicate an increase and decrease, respectively, of proteins between treatments.

**Figure 5:**
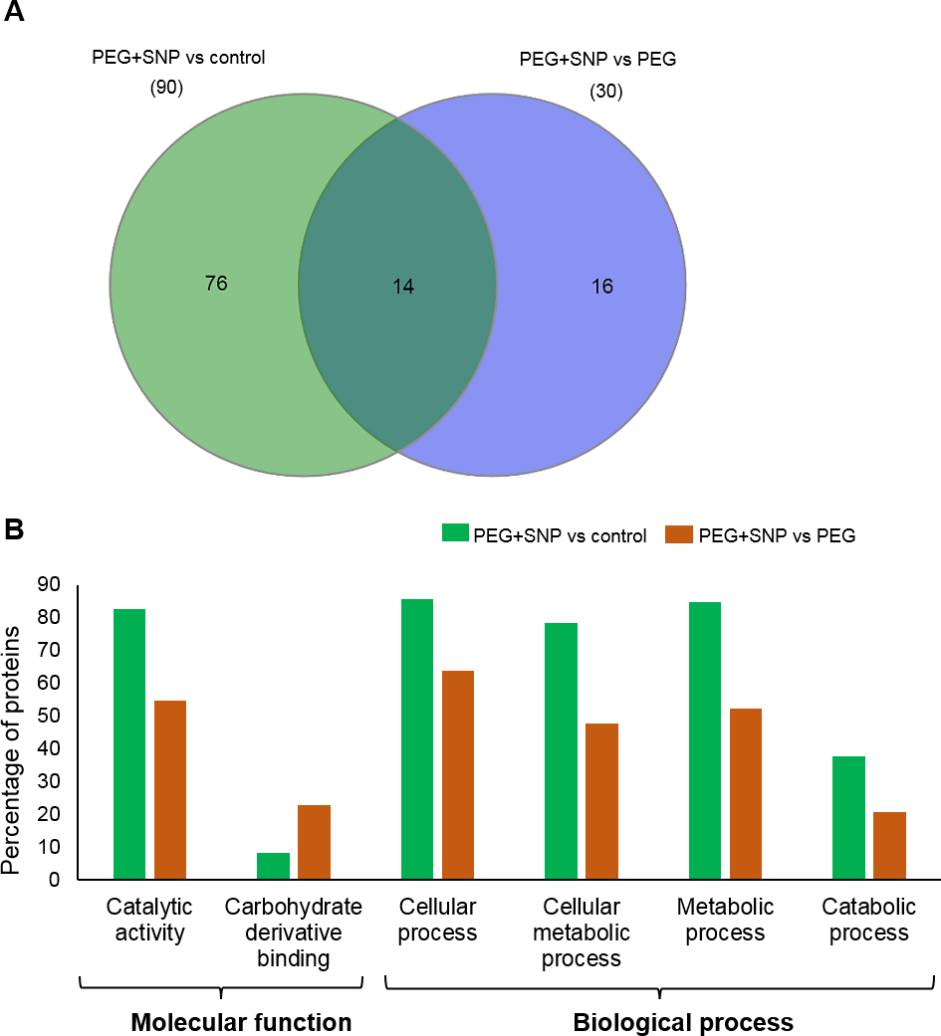
Functional categorization of identified proteins in the root of banana under osmotic stress. Proteins extracted from roots were identified using a gel-free proteomic technique and significantly changed proteins were compared. (A) The Venn diagram represented the comparison of proteins identified in the roots of banana treated with PEG with SNP against control or PEG. (B) Gene ontology category analysis of the protein’s comparison between PEG+SNP against control and PEG+SNP against PEG-treated banana. Proteins with differential abundance were classified as biological and molecular functions by WEGO according to the GO terms. The *x*-axis indicates the percentage of proteins. Any intensity showing statistical significance (*p* < 0.05) was considered to be positive.

To identify the protein changes of banana under osmotic stress, the total protein from the untreated root (control), root treated with PEG alone (PEG) or PEG and SNP (PEG+SNP) for 9 days were extracted for MS analysis. The data of all samples from different groups were compared by principal component analysis to determine the protein expression patterns from different treatments (Fig. S2). The identified proteins were used to generate heat maps (Fig. 4, Fig. S3). In total, 50 proteins differentially accumulated between PEG-treated banana and PEG and SNP treated banana (Fig. S3, Tables S1 and S2). Of these, 30 proteins were identified and categorized based on GO classification (Fig. 4). Totally 150 proteins differentially accumulated in SNP and PEG treatment against control. Among these, 90 proteins were identified (Fig. 5A).

The Venn diagram showed that 14 proteins similarly accumulated in both comparisons, whereas 76 and 16 proteins uniquely accumulated in PEG+SNP against control and PEG+SNP against PEG, respectively (Fig. 5A). Most of these proteins were responsible for cellular and metabolic processes (Fig. 5B). Based on the molecular function, 82.6% and 54.5% proteins involved in catalytic activity accumulated in comparison of PEG+SNP against control and PEG+SNP against PEG, respectively. Only 8.0% and 22.7% of proteins involved in carbohydrate derivative binding accumulated in comparison of PEG+SNP against control and PEG+SNP against PEG, respectively (Fig. 5B). When comparing the treatments based on biological function, the percentage of proteins for PEG+SNP against control was generally higher than PEG+SNP against PEG. Those proteins were mainly involved in cellular, cellular metabolic, metabolic, and catabolic processes (Fig. 5B).

**Figure 6:**
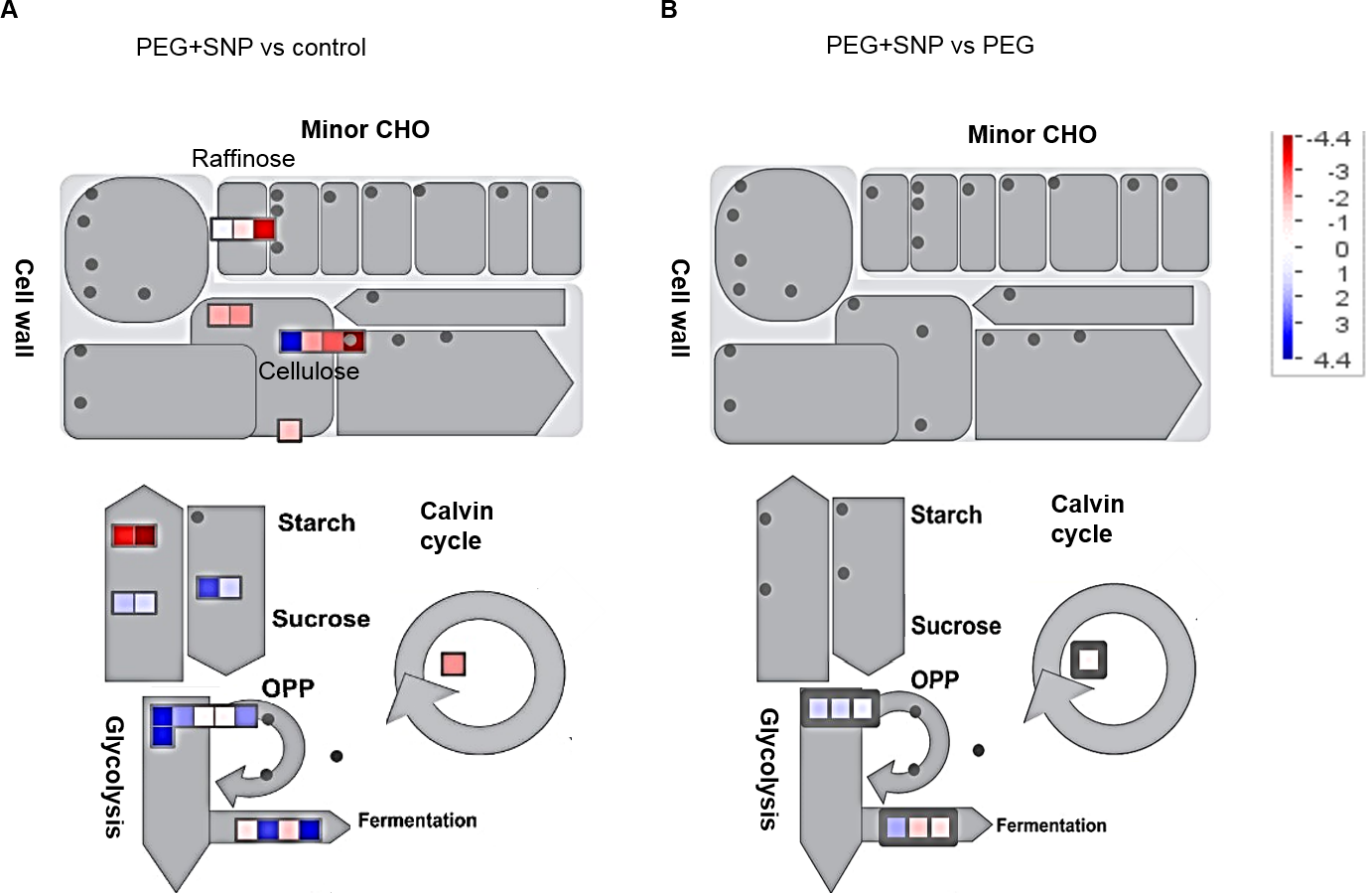
Abundances of proteins identified in banana roots treated with PEG with or without SNP. The fold change of proteins grouped into functional categories related to primary metabolism was mapped using MapMan software. The functional categories of the primary metabolic pathways of glycolysis and the TCA cycle are shown. Each square and color indicate the fold change value of a differentially changed protein. Red and blue colors indicate a decrease and increase, respectively, in fold change values compared with untreated and PEG-treated bananas. Abbreviations: CHO, carbohydrate; and OPP, oxidative pentose phosphate.

The identified proteins for each treatment were mapped into functional groups via MapMan (Fig. 6). In general, several proteins involved in pathways, such as oxidative pentose phosphate, fermentation, nitrate, one-carbon metabolism, and nucleotide activity, were found in all treatments. Other proteins involved in secondary metabolism, cell wall, and starch function accumulated in PEG+SNP-treated samples when compared to control (Fig. 6). The PEG+SNP-treated samples also showed enhanced protein accumulation in mitochondrion electron transport compared to PEG-treated samples (Fig. 6).

When integrating these proteins onto metabolic pathway using KEGG databases, the majority of proteins found in this study were related to the glycolysis process, such as glucose-6-phosphate isomerase (GPI), fructose-biphosphate aldolase (FBA), phosphoglycerate kinase (PGK), pyruvate kinase (PYK) and pyruvate dehydrogenase (PDH). Numerous proteins involved in the regulation of amino acid biosynthesis, such as cysteine synthase and D-3-phosphoglycerate dehydrogenase, tricarboxylic acid (TCA) cycle, such as malate dehydrogenase (MDH) and isocitrate dehydrogenase (IDH), as well as cell wall modification, such as both acetyl-CoA carboxylase and butyryl-CoA dehydrogenase, were also determined (Fig. S4).

GPI, FBA, PGK, enolase, and PDH were increased in abundance in comparison to PEG+SNP against control. When comparing PEG+SNP against PEG, the abundance of these proteins was reduced, except PYK and malic enzyme (Figs. 4 and 6).

### Antioxidant enzyme changes in PEG+SNP-treated and osmotic stressed bananas

**Figure 7:**
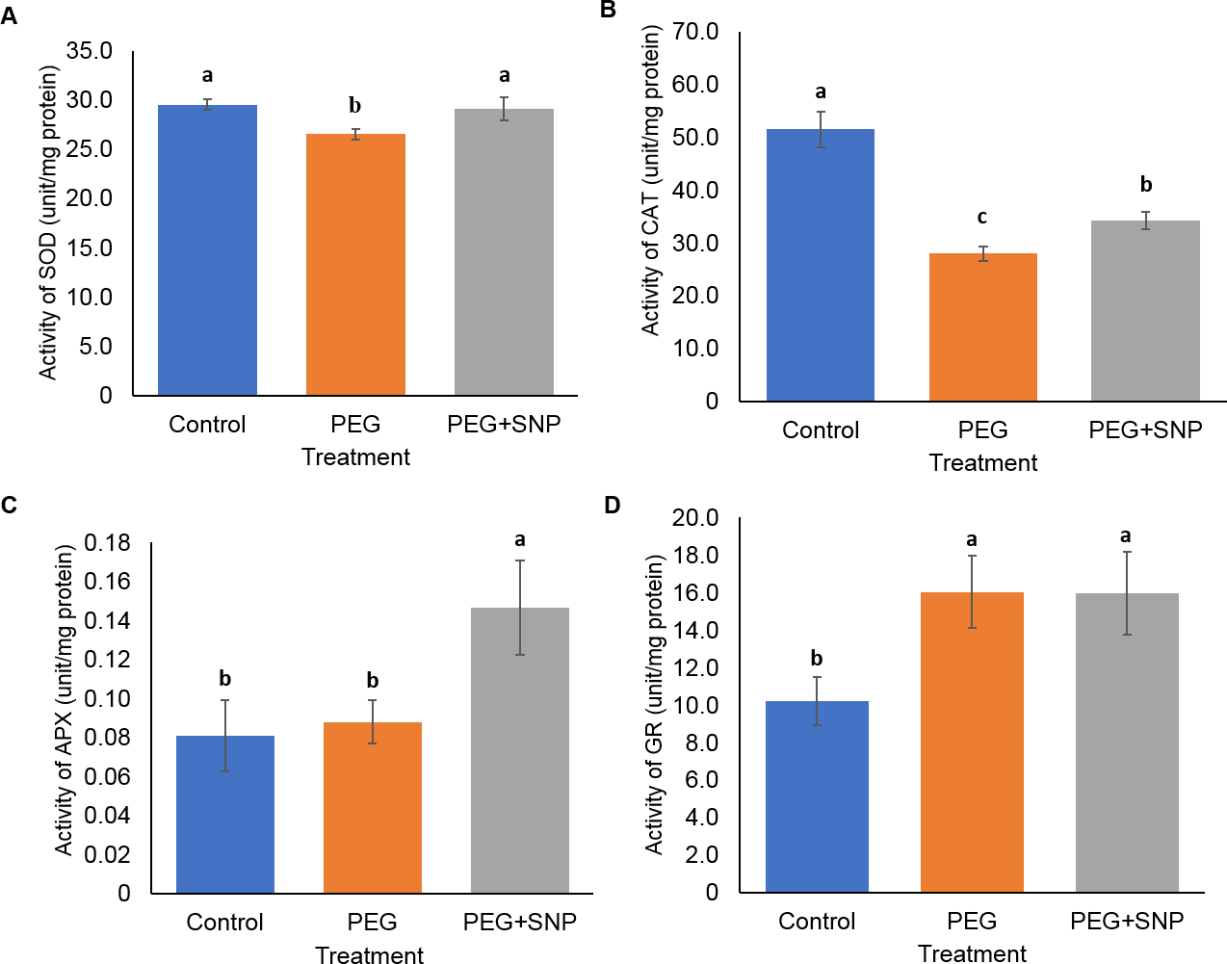
Antioxidant assays for banana leaves treated with PEG and with or without SNP. Activities of (A) SOD, (B) CAT, (C) APX, and (D) GR were measured through the extraction of banana leaves in phosphate buffer. Absorbance was measured through a spectrophotometer. SOD, CAT, APX, and GR were shown in Unit mg^−1^ protein. Protein content was shown as mg g^−1^ dry weight of leaf tissue. Data are shown as the means ± SE from three independent biological replicates. Means indicated with the same letter were not significantly different based on analysis of variance (ANOVA) followed by Duncan’s multiple range test at *p* < 0.05.

For leaf samples, the level of SOD for control and PEG+SNP (29.1–29.5 U mg^−1^) was significantly higher than PEG (26.5 U mg^−1^) (Fig. 7A). Under the SNP and PEG combination, the activity of CAT and APX was enhanced. The CAT activity of leaf samples was the highest (51.5 U mg^−1^) in well-watered bananas (control) followed by PEG+SNP (34.3 U mg^−1^) and PEG alone (28.0 U mg^−1^) (Fig. 7B). For APX, PEG+SNP-treated leaf samples showed higher (0.15 U mg^−1^) compared to well-watered banana (0.08 U mg^−1^) (Fig. 7C). PEG-treated and PEG+SNP leaf samples produced higher GR activity (16.0 U mg^−1^) compared to well-watered banana (10.2 U mg^−1^) (Fig. 7D).

**Figure 8:**
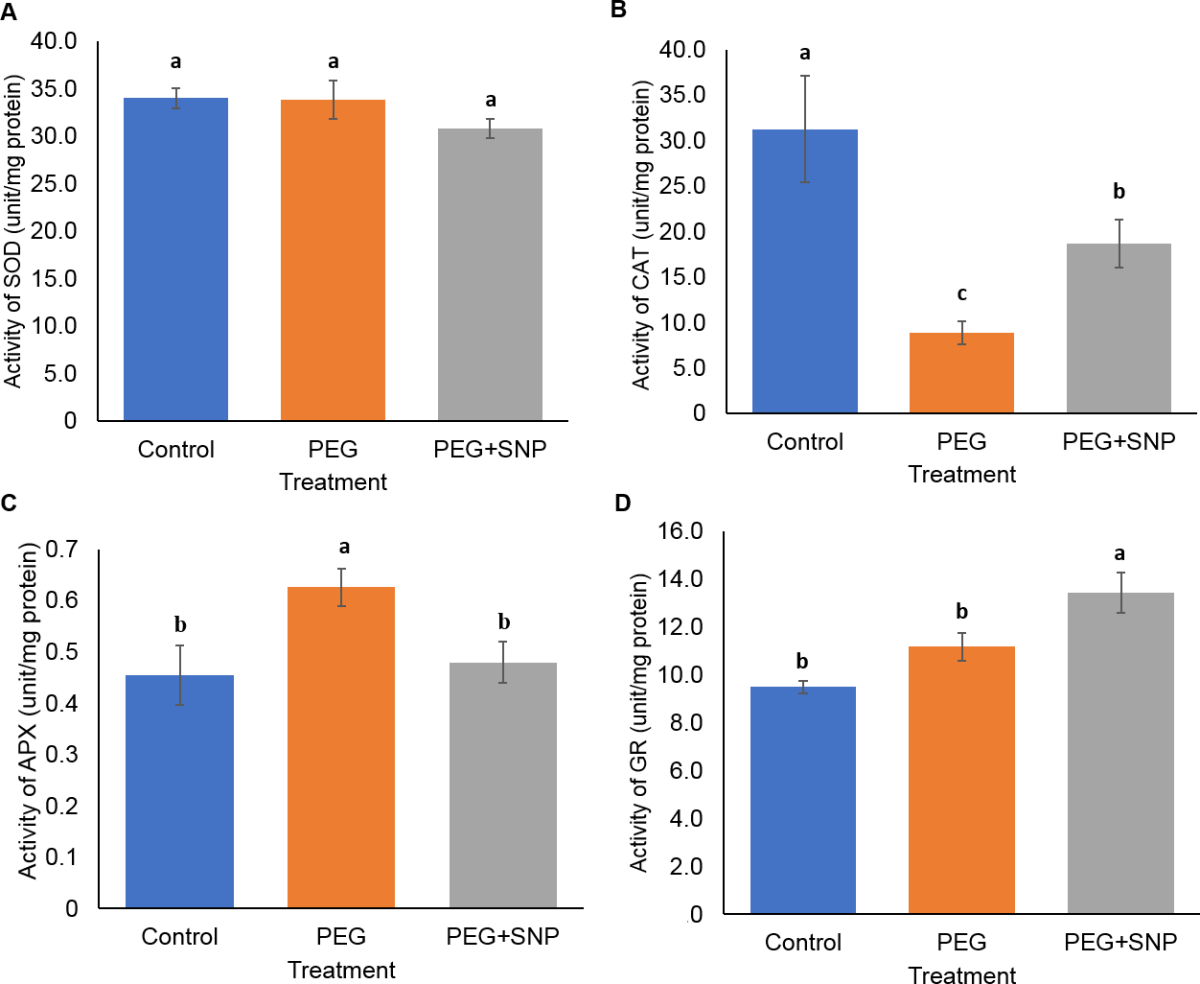
Antioxidant assays for banana roots treated with PEG and with or without SNP. Activities of (A) SOD, (B) CAT, (C) APX, and (D) GR were measured through the extraction of banana roots in phosphate buffer. Absorbance was measured through a spectrophotometer. SOD, CAT, APX, and GR were shown in Unit mg^−1^ protein. Protein was shown as mg g^−1^ dry weight of root tissue. Data are shown as the means ± SE from three independent biological replicates. Means indicated with the same letter were not significantly different based on analysis of variance (ANOVA) followed by Duncan’s multiple range test at *p* < 0.05.

The level of SOD in control (34.03 U mg^−1^) and PEG-treated (33.80 U mg^−1^) root samples was higher than PEG+SNP-treated samples (30.80 U mg^−1^) (Fig. 8A). Similarly to the leaf samples, PEG-treated root samples showed the least CAT activity (8.9 U mg^−1^) compared to the control (31.3 U mg^−1^) as well as PEG+SNP (18.7 U mg^−1^) (Fig. 8B). The APX activity of PEG-treated root samples, however, was higher (0.63 U mg^−1^) than control (0.46 mg^−1^) and PEG+SNP (0.48 U mg^−1^) (Fig. 8C). The GR activity for PEG+SNP-treated root samples was significantly higher than control and PEG-treated samples (Fig. 8D). Taken together, these results showed that the activity of CAT and APX in both leaves and roots was influenced by SNP treatment.

## Discussion

### Morphological alterations of PEG+SNP-treated and osmotic-stressed bananas

The growth and yield of banana are adversely affected by osmotic stress. In this study, SNP, which is a nitric oxide donor, was applied to PEG-treated bananas to investigate the morphological, biochemical, and protein changes of bananas in response to osmotic stress. The total dry weight of PEG-treated banana was strongly repressed. This reduction of growth might be due to the loss of cell turgor resulted from the increase of solutes concentration (Al-Yasi et al., 2020). The SNP application to the PEG-treated banana did not improve the leaf RWC. This contrasts with previous work, where nitric oxide improved RWC of plants under water stress conditions (Hajihashemi, 2020). One possible explanation for this finding is that the action of nitric oxide might depend on its concentration and on the plants analyzed. Bananas treated with SNP mitigated deleterious effects of osmotic stress by enhancing growth with an improved root formation and decreased leaf yellowing compared to PEG-treated bananas. Several plants treated with nitric oxide were found to produce a higher number of roots compared to the control. These include cucumber (Xu et al., 2017), trifoliate orange (Tian et al., 2017), and rice (Kushwaha et al., 2019). The involvement and requirement of nitric oxide in root development are generally accepted (Correa-Aragunde et al., 2016). Emerging evidence indicates that nitric oxide interacts with auxin in the regulation of lateral root development but inhibits root elongation (Sun et al., 2017), indicating that the relations between nitric oxide and auxin in regulating root growth are complex. Nitric oxide has also been shown to interact with cytokinin and ascorbic acid to regulate cell division during the de-differentiation and re-differentiation of plant cells (Tan, Chin & Alderson, 2013; Sun et al., 2018).

Nitric oxide is also an important modulator in osmotic tolerance and senescence Shi et al. (2014). In this study, leaf yellowing in PEG-treated banana was reversed by the addition of SNP. Leaf senescence due to osmotic stress is usually accompanied by down-regulation of photosynthesis-related genes and reduced chlorophyll content (Kong et al., 2016). This is because plants are relocating nutrients from older senescing leaves to younger organs. As a result, leaves become yellow due to a loss of chlorophyll (Bruand & Meilhoc, 2019). Exogenous application of SNP could delay leaf senescence by inhibiting chlorophyll degradation (Liu & Guo, 2013). Plants grown under drought conditions increased the accumulation of nitric oxide to prevent water loss via ABA-induced stomatal closure (Yang et al., 2018). Applying the nitric-oxide scavenger, 2-phenyl-4,4,5,5,-tetramethylimidazoline-1-oxyl 3-oxide (PTIO), however, inhibited stomatal closure. This supports that nitric oxide is induced endogenously and is most likely the key signaling element facilitating stomatal closure (Yang et al., 2018).

### Carbohydrate and energy metabolism-related protein abundance under osmotic stress

Plants adapt to osmotic stress conditions by changing genes and reorganizing metabolic pathways and physiological processes to improve their survival (Tan et al., 2013; Yeganehpoor et al., 2016). In this study, a label-free proteomics approach was used to analyze protein changes in response to osmotic stress in banana. This technique has been used to examine plasma membrane proteins obtained from osmotic stressed-soybean seedlings (Nouri & Komatsu, 2010) and seed proteins from wheat subjected to PEG, salinity, and submergence treatments (Yan et al., 2020). Proteins involved in metabolic and energy processes were identified as the largest differentially changed protein group in this study, demonstrating their important roles in response to osmotic stress conditions. Under osmotic stress, plants alter their carbohydrate metabolic processes to produce soluble sugars that regulate plant-cell osmotic conditions and cell-wall integrity (Zhang et al., 2017a; Zhang et al., 2017b). In osmotic-stressed banana (PEG+SNP), glycolytic enzymes and proteins involved in carbohydrate metabolism were increased in abundance, most probably due to energy demand caused by osmotic stress. Six of the proteins of these classes are GPI, FBA, PGK, PYK, PDH, and IDH. GPI has been shown to have high abundance in plants under severe water stress, such as *Platycladus orientalis* (Zhang et al., 2017a; Zhang et al., 2017b) and wheat (Xue et al., 2008). Under the osmotic condition, carbohydrate is massively accumulated and starch breakdown by several enzymes, including GPI, with a concomitant increase of soluble sugars, is often observed (Praxedes et al., 2006). Taken together, the present and previous findings suggest that increased glycolytic activity is needed to meet the energy demands in osmotic tolerance.

FBA interacts with ABA and other stress signaling molecules in plants (Cho & Yoo, 2011). Studies showed that the mRNA level and enzyme activity of FBA in plants were increased in response to abiotic stress (Fan, Zhang & Zhang, 2009). In this study, the abundance of FBA in comparison of PEG+SNP against PEG was reduced compared to PEG+SNP against control, suggesting the regulation function of nitric oxide in osmotic tolerance. The accumulation of FBA in osmotic-stressed banana might be an adaptation of plants to water deficit environment by producing more energy and dissipating the metabolites efficiently via trans-localization to maintain cell metabolism. Enolase was increased in roots of banana treated with PEG+SNP, but the addition of SNP could reduce its accumulation. Enolase is a metalloenzyme that catalyzes the conversion of 2-phosphoglycerate (2-PG) into PEP in the glycolysis pathway (Çevik et al., 2019). Its great abundance shown by other researchers in different osmotic-stressed plants (Oh & Komatsu, 2015) could be due to the need of cells for extra energy to cope with stress and repair damage (Wang et al., 2016). Moreover, modification of cysteines between Cys318 and Cys346 in enolase by nitric oxide might have initiated S-nitrosylation to regulate cellular processes, including posttranslational modification (Dumont & Rivoal, 2019). These findings support the protective role of nitric oxide in preventing cellular oxidative stress.

PDH increased in PEG+SNP treated bananas compared to control, suggesting a possible increased flux of carbohydrates to the TCA cycle. This would provide more energy for plants to cope with osmotic stress. In the present study, PDH was decreased under SNP treatment, which implied that less energy needed for plants to cope with osmotic stress. When comparing drought-sensitive and tolerant barley, PDH was abundantly accumulated on both sensitive and tolerant barleys, suggesting the important role of PDH in enhancing osmotic tolerance (Guo et al., 2009). The amounts of PDH were also increased in drought-stressed foxtail millet (Pan et al., 2018). However, the conversion of pyruvate to acetyl-CoA by PDH in the glycolysis activity was inhibited by nitric oxide in Arabidopsis, leading to a decrease in cellular glycolysis enzymes, ATP synthase activities, and acetyl coenzyme A (Zhang et al., 2017a; Zhang et al., 2017b). These findings suggest that PDH responds to nitric oxide and may be an important enzyme for shifting energy metabolism in the roots of banana under osmotic condition.

IDH is a nicotinamide adenine dinucleotide phosphate (NADP+)-dependent dehydrogenase which catalyzes the oxidative decarboxylation of isocitrate to *α*-ketoglutarate. Under abiotic stress conditions, an additional NADPH supply may be required. Evidence indicates that one or more NADP-dehydrogenases are produced at gene or protein levels under stress conditions (Corpas & Barroso, 2014). IDH has been shown to be abundantly accumulated in grapevine variety M4 (Prinsi et al., 2018) and soybean (Wang & Komatsu, 2017) under stress condition. A similar finding has been reported in olive plants (Valderrama et al., 2006). All NADP-dehydrogenases such as glucose-6-phosphate dehydrogenase, 6-phosphogluconate dehydrogenase, NADP-malic enzyme, and NADP-isocitrate dehydrogenase, were increased under oxidative stress, suggesting that plants overcome any potential cellular damage by inducing NADP-dehydrogenases (Valderrama et al., 2006). These findings indicate the important role of IDH in response to abiotic stresses in plants.

PYK, which catalyzes the final step of glycolysis to convert phosphoenolpyruvate (PEP) and adenosine diphosphate to pyruvate and ATP, was increased under SNP treatment. The influence of nitric oxide on protein kinase activities has been poorly studied in plants. It has been shown to mediate osmotic-induced cyanide-resistant alternative pathway in wheat by increasing *AOX1a* expression and pyruvate content (Wang et al., 2016). The enhanced cyanide-resistant alternative pathways by nitric oxide could assist plants to reduce ROS production and oxidative damage. On the other hand, the addition of SNP increased the accumulation of malic enzyme, which is an essential enzyme metabolizing malate. Malic enzyme was suggested to be involved in various stress responses in plants (Chen et al., 2019).

Three MDHs were less abundantly accumulated in this study. MDH is a NAD-dependent enzyme that catalyzes malate into oxaloacetate in reversible oxidation to form NADH (Liu & Guo, 2013). Although MDH has been reported to be abundantly accumulated in both soil drought and PEG stress conditions (Cui et al., 2019), the abundance of MDH might be influenced by the variety of plants (Kausar et al., 2013). Kausar et al. (2013) demonstrated that the drought susceptible potato variety produced less MDH compared to the drought-tolerant variety. The less abundance of MDH in this study might be also due to the susceptibility of this cultivar to osmotic condition. These results suggested that MDH has an important role in tolerance to osmotic stress but showed different responses in tolerant and sensitive genotypes. Other proteins, such as glycoside hydrolase and short-chain dehydrogenase/reductase, were less abundantly accumulated for PEG+SNP against PEG compared to PEG+SNP against control. These findings suggest that PEG-induced osmotic stress enhances the accumulation of proteins involved in metabolic and energy processes, but the application of SNP could reduce the accumulation of these proteins.

### Nitric oxide altered the production of ROS

High accumulation of ROS severely impacts plant growth and development. Plants regulate the level of ROS through antioxidant enzyme systems to protect them from destructive oxidative reactions. In this study, SOD activity in control and PEG+SNP-treated leaves was higher than PEG-treated, while it had no significant difference in roots. APX activity was the highest in the PEG-treated root. However, CAT activity was the lowest in the PEG-treated root. This might be due to the higher affinity of APX for H_2_O_2_ compared to CAT (Aziz et al., 2017). APX and CAT activities in both leaves and roots were influenced by SNP treatment. In sugarcane, spraying S-nitrosogluthatione, which is a nitric oxide donor, resulted in increases of CAT under water deficit (Silveira et al., 2016). The application of SNP enhanced the CAT activity in PEG-treated rice, whereas PEG treatment alone reduced the CAT activity (Cao et al., 2019). Given its effects on maintaining ROS homeostasis, it is possible that nitric oxide could improve plant-osmotic tolerance by activating the antioxidant-defense systems. Exogenous application of nitric oxide alleviated osmotic stress through increasing the ROS scavenging enzymes, proline, and osmolyte metabolism in many plant species, such as *Oryza sativa* (Farooq et al., 2009), *Poncirus trifoliate* (Fan & Liu, 2012), and *Tagetes erecta* (Liao et al., 2012). The levels of non-enzymatic antioxidant and antioxidant enzyme activities in PEG+SNP treated- rapeseed and wheat seedlings were enhanced (Hasanuzzaman et al., 2018). This molecule could also enhance the levels of other antioxidant molecules like flavonoids, glutathione, and ascorbate to scavenge the harmful ROS (Li et al., 2017). Overall, the present and previous results demonstrate that exogenous nitric oxide may increase plant antioxidant capacity and help plants to survive under stress conditions. Nonetheless, the interaction among nitric oxide, hormone signaling pathways, and the molecular mechanisms involved in this process is likely to be discovered.

## Conclusions

In summary, a combined analysis of plant morphology, antioxidant enzyme activities, and the root proteome in banana clearly showed that the PEG-induced osmotic stress negatively affected the banana plants. The decline in RWC, total dry weight, and number of roots were the most noticeable differences between stressed and non-stressed banana plants. The supplementation of SNP to osmotic-stressed banana plants, however, improved their total weight, root formation, and reduced leaf yellowing symptom. The mechanism by which nitric oxide enhances the osmotic stress tolerance in PEG-treated banana was determined both at the proteome and antioxidant levels. The majority of proteins differing between osmotic-stressed and SNP-treated osmotic-stressed bananas were classified as carbohydrate and energy metabolism. APX and CAT activities in both leaves and roots were also influenced by SNP treatment. The main findings of this study are: (i) the growth of banana was adversely affected by PEG-induced osmotic stress; (ii) exogenous application of SNP mitigated deleterious effects of PEG-induced osmotic stress by enhancing root formation and decreasing leaf yellowing; and (iii) glycolysis and energy metabolism-related proteins were altered in response to SNP under osmotic stress. These findings suggest that nitric oxide has an important role in the acclimation of banana to water stress through the regulation of glycolysis and redox related pathways. Certainly, further efforts in future works, such as quantitative PCR assays, should be performed to verify the conclusion.

##  Supplemental Information

10.7717/peerj.10879/supp-1 Figure S1 Pictures of the three groups according to their color, which are green, green/yellow, and yellow/brown

10.7717/peerj.10879/supp-2 Figure S2 Overview of total proteomic data from 18 samples based on principle component analysis

10.7717/peerj.10879/supp-3 Figure S3 Cluster analysis of osmotic-responsive proteins induced by PEG and SNP in banana

10.7717/peerj.10879/supp-4 Figure S4 Mapping of differentially changed proteins that related to carbon metabolism identified from PEG+SNP against control and PEG+SNP against PEG-treated banana roots Image credit: Kanehisa Laboratories.

10.7717/peerj.10879/supp-5 Supplemental Information 5 Abundance of identified proteins in roots of banana treated with SNP under PEG-treated samples compared to control and PEG-treated samples

10.7717/peerj.10879/supp-6 Supplemental Information 6 Raw data for the morphological changes after PEG treatment Data for the percentage of yellow leaves, total weight and the number of newly formed roots.

10.7717/peerj.10879/supp-7 Supplemental Information 7 Raw data for antioxidant assays Antioxidant assays for banana leaves and roots treated with PEG and with or without SNP under osmotic stress.

## Additional Information and Declarations

### Data Availability

The following information was supplied regarding data availability: Raw data is available at the ProteomeXchange Consortium: PXD017689.
