# Supplementary figures and images for: Osmotic stress in banana is relieved by exogenous nitric oxide
Source: PeerJ. 2021 Feb 9;9:e10879. doi: 10.7717/peerj.10879 (PMC7879939; doi:10.7717/peerj.10879)

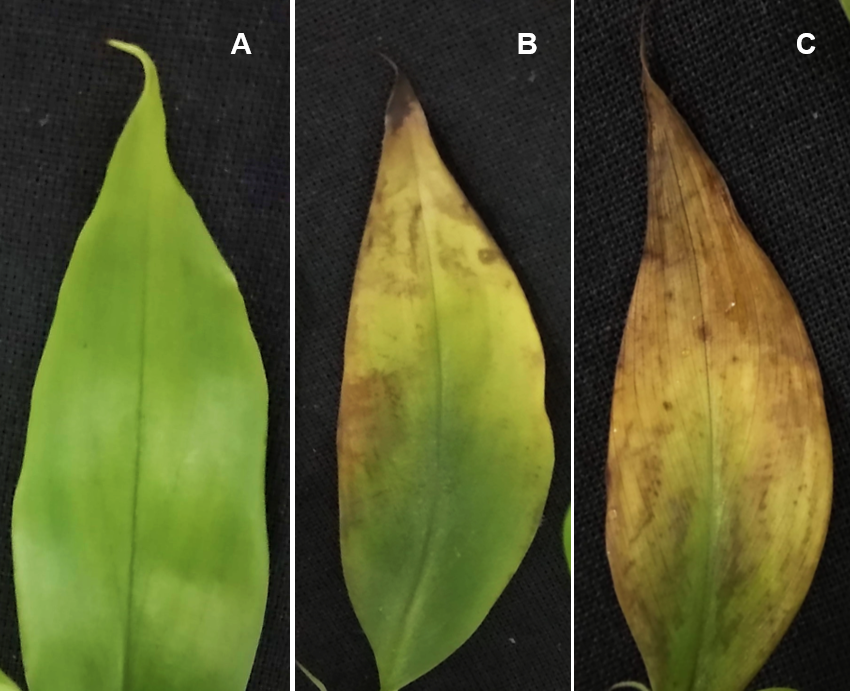

Supplement: Figure S1 [file peerj-09-10879-s001.png]

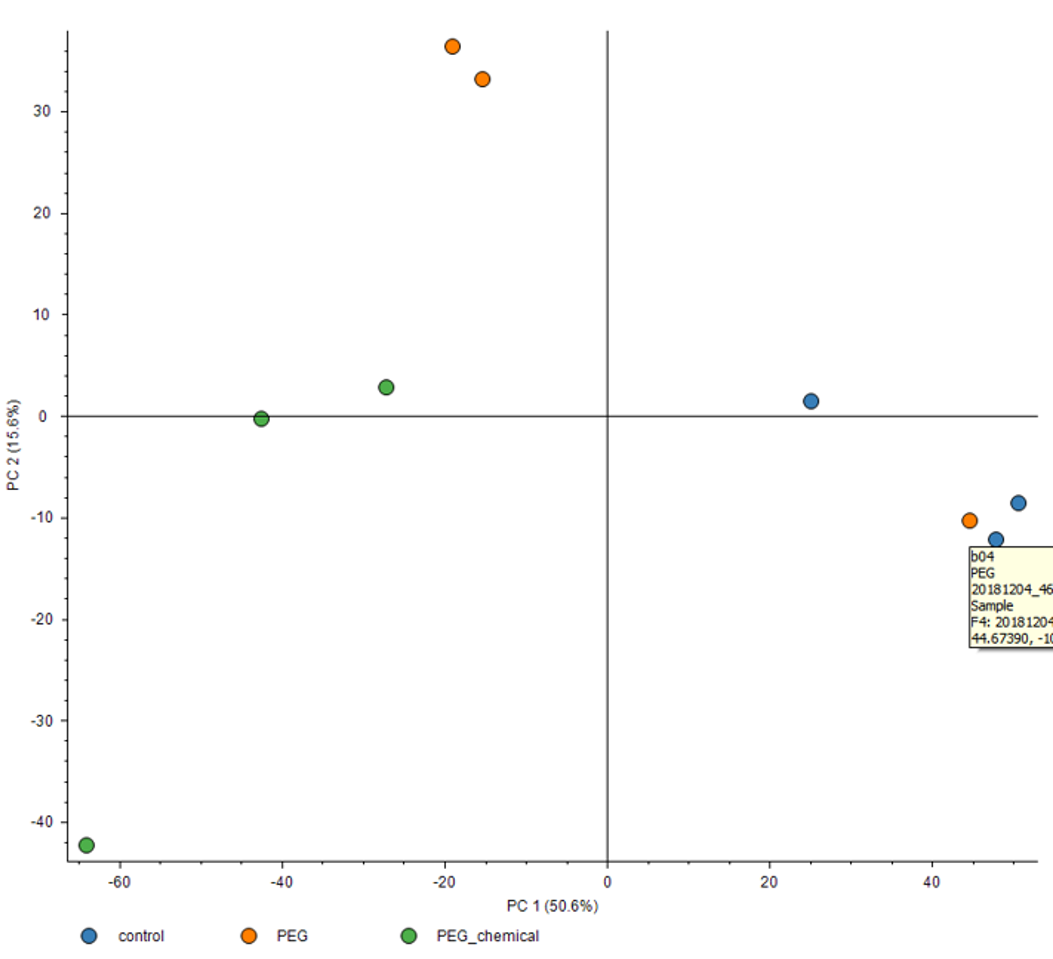

Supplement: Figure S2 [file peerj-09-10879-s002.png]

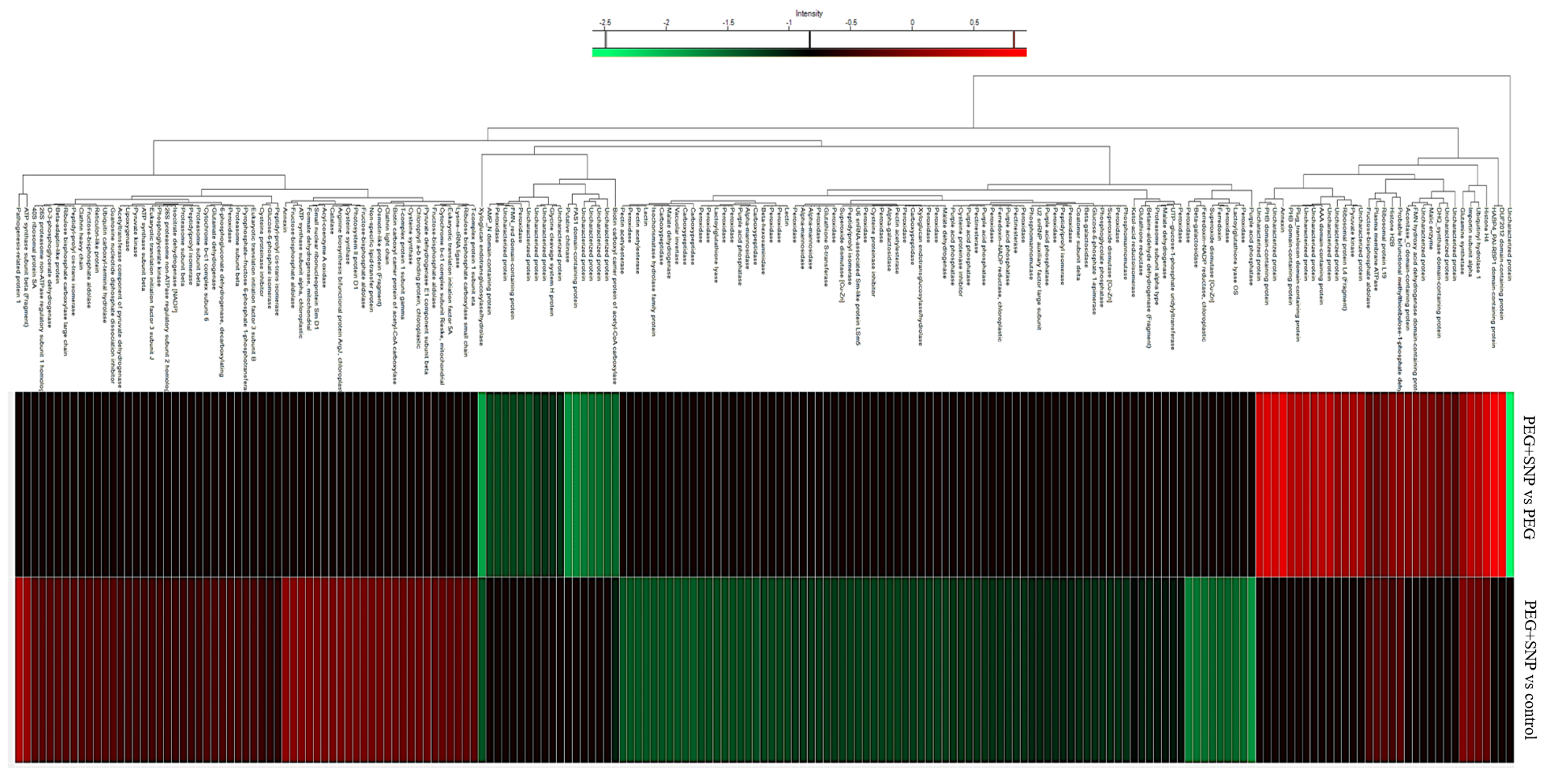

Supplement: Figure S3 [file peerj-09-10879-s003.png]

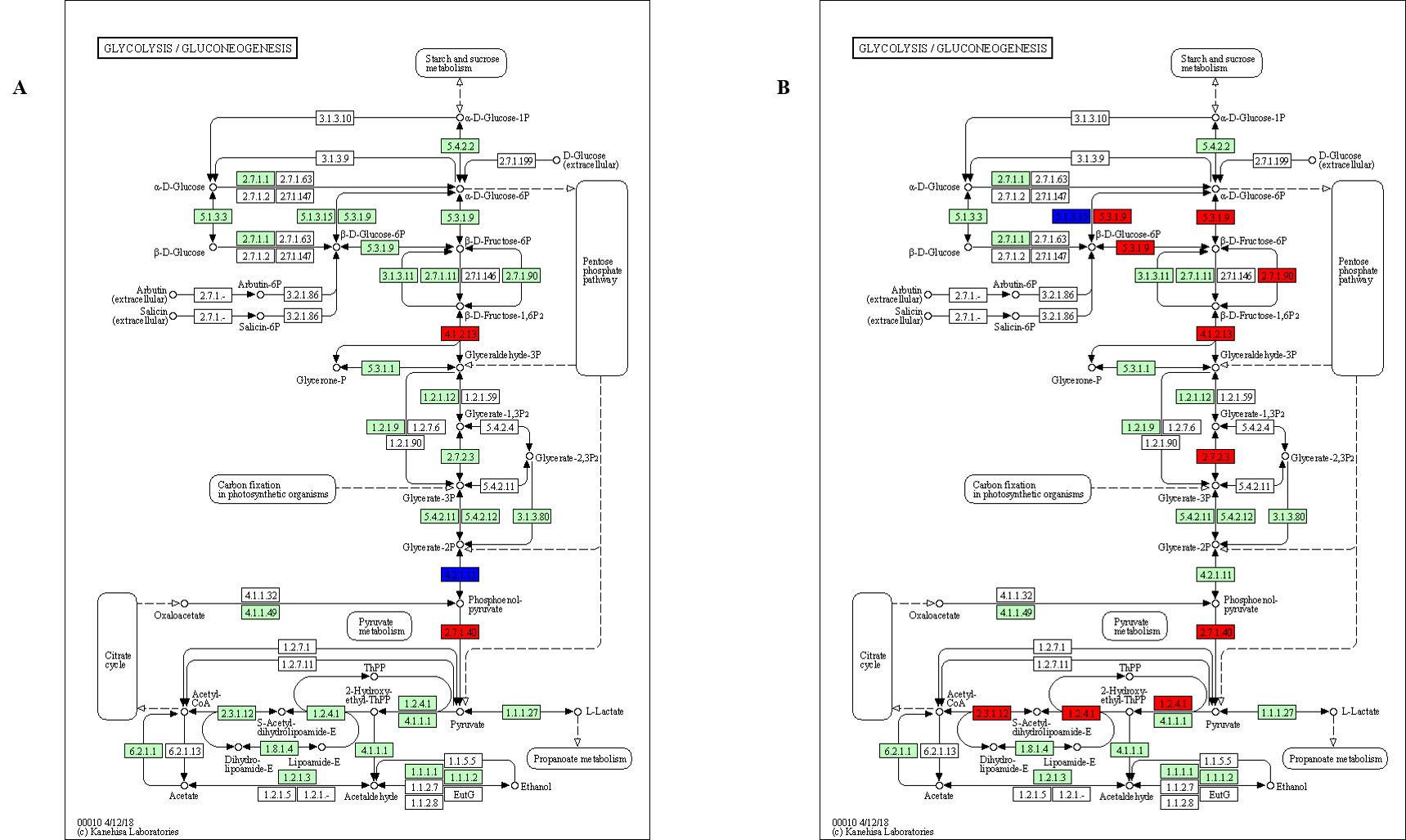

Supplement: Figure S4 — Image credit: Kanehisa Laboratories. [file peerj-09-10879-s004.png]
