# Supplementary material for: Osmotic stress in banana is relieved by exogenous nitric oxide
Source: PeerJ. 2021 Feb 9;9:e10879. doi: 10.7717/peerj.10879 (PMC7879939; doi:10.7717/peerj.10879)
Supplement: Supplemental Information 5 [file peerj-09-10879-s005.docx]

Supplementary Table 1. Protein changes of root treated with PEG+SNP against control.

| Protein names | Protein IDs | # Unique peptides | Q-Value | Relative abundance  (PEG+SNP/control) |
| --- | --- | --- | --- | --- |
| Pathogenesis-related protein 1 | W0FE88 | 5 | 2.74154917 | 5.445 |
| ATP synthase subunit beta (Fragment) | A9QBI7 | 18 | 2.17628252 | 4.455 |
| Annexin | M0SCM5 | 3 | 2.1759066 | 4.075 |
| Fructose-bisphosphate aldolase | M0T7Y8 | 6 | 3.16715969 | 3.831 |
| ATP synthase subunit alpha, chloroplastic | S6E0P0 | 11 | 1.90635461 | 3.732 |
| Glutamine synthetase | M0U5F8 | 3 | 2.03288821 | 3.706 |
| Formate dehydrogenase, mitochondrial | M0SAT8 | 11 | 1.71031003 | 3.609 |
| Small nuclear ribonucleoprotein Sm D1 | M0SMJ1 | 2 | 3.45702474 | 3.578 |
| Acyl-coenzyme A oxidase | M0TFI4 | 11 | 2.09877875 | 3.528 |
| Catalase | M0TQ39 | 8 | 2.25656768 | 3.441 |
| Arginine biosynthesis bifunctional protein ArgJ, chloroplastic | M0RHY2 | 3 | 2.06650411 | 3.422 |
| Cysteine synthase | M0U3I3 | 8 | 1.92464541 | 3.395 |
| Photosystem II protein D1 | S6E0N4 | 2 | 2.19013911 | 3.332 |
| Fructose-bisphosphate aldolase | M0TNG5 | 3 | 2.26002878 | 3.330 |
| Importin subunit alpha | M0SHD1 | 9 | 1.70573626 | 3.235 |
| Non-specific lipid-transfer protein | M0SPH7 | 2 | 2.01308518 | 3.211 |
| Osmotin-like protein (Fragment) | Q8LKA8 | 2 | 2.26847686 | 3.176 |
| Clathrin light chain | M0T0A6 | 4 | 2.05849043 | 3.172 |
| Ubiquitinyl hydrolase 1 | M0TW49 | 4 | 1.6099014 | 3.098 |
| Biotin carboxyl carrier protein of acetyl-CoA carboxylase | M0TLK7 | 4 | 3.04008465 | 2.991 |
| T-complex protein 1 subunit gamma | M0T9H7 | 5 | 1.68997378 | 2.955 |
| Cysteine synthase | M0SZX7 | 13 | 2.01460502 | 2.938 |
| Chlorophyll a-b binding protein, chloroplastic | Q1EP00 | 5 | 2.18012136 | 2.927 |
| Pyruvate dehydrogenase E1 component subunit beta | M0T1H5 | 7 | 1.95941084 | 2.915 |
| Fructose-bisphosphate aldolase | M0TR81 | 5 | 2.12141887 | 2.793 |
| Cytochrome b-c1 complex subunit Rieske, mitochondrial | M0SG26 | 3 | 1.55272503 | 2.722 |
| Histone H4 | M0RV37 | 4 | 1.74169184 | 2.677 |
| Eukaryotic translation initiation factor 5A | M0RJA0 | 2 | 2.32452342 | 2.658 |
| Lysine--tRNA ligase | M0RM50 | 3 | 1.71262678 | 2.584 |
| Ribulose bisphosphate carboxylase small chain | A5JPN4 | 3 | 2.00053018 | 2.576 |
| T-complex protein 1 subunit eta | M0TR12 | 10 | 2.2929789 | 2.502 |
| 40S ribosomal protein SA | M0S1Z0 | 3 | 1.84968429 | 2.410 |
| 26S proteasome non-ATPase regulatory subunit 1 homolog | M0RYR4 | 13 | 1.98164867 | 2.393 |
| Probable bifunctional methylthioribulose-1-phosphate dehydratase/enolase-phosphatase E1 | M0TAQ9 | 6 | 2.51427124 | 2.362 |
| D-3-phosphoglycerate dehydrogenase | M0U2N8 | 4 | 1.51447261 | 2.355 |
| Plasma membrane ATPase | M0RYP4 | 10 | 1.85643575 | 2.347 |
| Beta-adaptin-like protein | M0S2M7 | 7 | 1.59115707 | 2.282 |
| Ribulose bisphosphate carboxylase large chain | S6DES6 | 9 | 1.69910485 | 2.259 |
| Peptidyl-prolyl cis-trans isomerase | M0TIB9 | 2 | 1.71373649 | 2.256 |
| Ribosomal protein L19 | M0U1Z1 | 2 | 1.70015167 | 2.234 |
| Clathrin heavy chain | M0SWT1 | 3 | 1.58165328 | 2.183 |
| Fructose-bisphosphate aldolase | M0U9P4 | 5 | 1.89523082 | 2.155 |
| Reticulon-like protein | M0RKC5 | 2 | 1.57734048 | 2.127 |
| Ubiquitin carboxyl-terminal hydrolase | M0RUN0 | 3 | 1.72959305 | 2.126 |
| Guanosine nucleotide diphosphate dissociation inhibitor | M0T836 | 5 | 1.66638404 | 2.119 |
| Cytochrome b-c1 complex subunit 6 | M0SUL0 | 2 | 2.74646773 | 1.975 |
| Glutamate dehydrogenase | M0S6T4 | 8 | 2.81015209 | 1.932 |
| 6-phosphogluconate dehydrogenase, decarboxylating | M0SF31 | 8 | 2.69002367 | 1.853 |
| Peroxidase | M0TPT9 | 10 | 1.70578346 | 1.828 |
| Proteasome subunit beta | M0T186 | 9 | 1.86178945 | 1.825 |
| Pyrophosphate--fructose 6-phosphate 1-phosphotransferase subunit beta | M0TSR2 | 7 | 1.64718585 | 1.812 |
| Eukaryotic translation initiation factor 3 subunit B | M0RN39 | 15 | 2.33360712 | 1.800 |
| Fructose-bisphosphate aldolase | M0T5K0 | 5 | 1.93839693 | 1.779 |
| Acetyltransferase component of pyruvate dehydrogenase complex | M0RGD1 | 11 | 1.82430103 | 1.717 |
| Lipoxygenase | M0U1N2 | 16 | 2.11124945 | 1.696 |
| Pyruvate kinase | M0RRM3 | 4 | 1.7635401 | 1.660 |
| Histone H2B | M0TCC5 | 2 | 2.82328567 | 1.643 |
| ATP synthase subunit beta | M0U2D0 | 3 | 1.82827184 | 1.633 |
| Eukaryotic translation initiation factor 3 subunit J | M0S2B1 | 5 | 2.22292351 | 1.569 |
| Phosphoglycerate kinase | M0SU77 | 4 | 2.02003507 | 1.566 |
| 26S proteasome non-ATPase regulatory subunit 2 homolog | M0RUP5 | 18 | 2.19097286 | 1.528 |
| Isocitrate dehydrogenase [NADP] | M0THE6 | 6 | 1.78733309 | 1.526 |
| Proteasome subunit beta | M0TYE4 | 9 | 1.64850432 | 1.465 |
| Peptidylprolyl isomerase | M0U5Q9 | 6 | 1.61853345 | 1.435 |
| Proteasome subunit beta | M0RGI2 | 3 | 1.86364556 | 1.390 |
| Cysteine proteinase inhibitor | M0SFR4 | 8 | 1.92102468 | 1.230 |
| Glucose-6-phosphate isomerase | M0TFQ1 | 11 | 2.67541547 | 1.101 |
| Peptidyl-prolyl cis-trans isomerase | M0SE30 | 6 | 2.22813527 | 0.922 |
| Ketol-acid reductoisomerase | M0TKD3 | 7 | 2.0941128 | -0.995 |
| Peroxidase | M0SL92 | 7 | 1.85494417 | -1.355 |
| Proteasome subunit alpha type | M0S4T8 | 4 | 1.98261156 | -1.489 |
| Malate dehydrogenase | M0SR05 | 9 | 1.80972852 | -1.504 |
| UTP--glucose-1-phosphate uridylyltransferase | M0U300 | 16 | 2.82676841 | -1.576 |
| Glutathione reductase | M0RYV2 | 12 | 1.8565511 | -1.874 |
| Betaine-aldehyde dehydrogenase (Fragment) | Q6DQ92 | 2 | 2.08050449 | -1.880 |
| Biotin carboxyl carrier protein of acetyl-CoA carboxylase | M0TKZ6 | 3 | 1.91847252 | -1.885 |
| Peptidylprolyl isomerase | M0TJW5 | 7 | 1.69140056 | -2.007 |
| Peroxidase | M0T946 | 6 | 2.37508457 | -2.059 |
| Phosphomannomutase | M0SI92 | 5 | 3.22269221 | -2.119 |
| U2 snRNP auxiliary factor large subunit | M0SMW7 | 4 | 1.60323719 | -2.146 |
| Purple acid phosphatase | M0S360 | 7 | 1.78358239 | -2.147 |
| Peroxidase | M0TEQ7 | 2 | 1.62149699 | -2.197 |
| Coatomer subunit delta | M0SG75 | 3 | 2.4429816 | -2.259 |
| Beta-galactosidase | M0SX47 | 13 | 1.86425085 | -2.292 |
| Glucose-6-phosphate 1-epimerase | M0SAP7 | 2 | 1.7786948 | -2.299 |
| Phosphoglycolate phosphatase | M0TUI6 | 4 | 1.75454163 | -2.311 |
| Superoxide dismutase [Cu-Zn] | A0A097I4Z6 | 3 | 2.52016966 | -2.333 |
| Peroxidase | M0TVH3 | 4 | 2.64594748 | -2.336 |
| Phosphomannomutase | M0SI93 | 3 | 2.47588931 | -2.349 |
| Peroxidase | M0TE60 | 5 | 2.52721299 | -2.424 |
| Ferredoxin--NADP reductase, chloroplastic | M0STS9 | 6 | 1.77573633 | -2.426 |
| Purple acid phosphatase | M0S7P5 | 11 | 1.83184084 | -2.452 |
| Pectinesterase | M0SUB7 | 10 | 2.22821916 | -2.490 |
| Peroxidase | M0T642 | 2 | 2.40724649 | -2.490 |
| Purple acid phosphatase | M0U9C6 | 2 | 1.77877902 | -2.552 |
| Cysteine proteinase inhibitor | M0RTU0 | 4 | 1.85975621 | -2.564 |
| Purple acid phosphatase | M0SU42 | 13 | 1.75002722 | -2.566 |
| Pectinesterase | M1RX38 | 2 | 1.58293636 | -2.582 |
| Purple acid phosphatase | M0U5B2 | 6 | 2.77629889 | -2.592 |
| Peroxidase | M0RI48 | 10 | 1.68480687 | -2.656 |
| Alpha-mannosidase | M0TWG0 | 23 | 1.84770967 | -2.708 |
| Alpha-mannosidase | M0U935 | 28 | 2.02515101 | -2.716 |
| Peroxidase | M0TBJ2 | 8 | 1.62049969 | -2.721 |
| Glutathione S-transferase | M4PXA4 | 2 | 1.66161142 | -2.724 |
| Peroxidase | M0SEE4 | 11 | 1.66069519 | -2.732 |
| Peroxidase | M0SYV4 | 8 | 1.8394156 | -2.794 |
| Carboxypeptidase | M0SWM7 | 11 | 2.02287166 | -2.799 |
| Xyloglucan endotransglucosylase/hydrolase | M0TRX7 | 4 | 2.37299058 | -2.819 |
| Peroxidase | M0RLG2 | 5 | 2.15691141 | -2.827 |
| Peroxidase | M0T1H9 | 4 | 1.56461299 | -2.837 |
| Malate dehydrogenase | M0U880 | 7 | 2.7339718 | -2.846 |
| Superoxide dismutase [Cu-Zn] | L7S115 | 2 | 1.80175943 | -2.875 |
| Peptidylprolyl isomerase | M0SVY4 | 3 | 2.76259182 | -2.903 |
| U6 snRNA-associated Sm-like protein LSm5 | M0TLE5 | 2 | 1.72087998 | -2.909 |
| Peroxidase | M0S9P2 | 3 | 1.96977333 | -2.920 |
| Cysteine proteinase inhibitor | M0SIZ8 | 3 | 1.80719601 | -2.924 |
| Peroxidase | M0U4Z8 | 8 | 1.77128827 | -2.951 |
| Alpha-galactosidase | M0T7D0 | 12 | 3.38515216 | -2.954 |
| Pectin acetylesterase | M0TQF6 | 11 | 2.18143028 | -2.959 |
| Peroxidase | M0T5C0 | 8 | 4.05399797 | -3.047 |
| Lectin | E9NX13 | 2 | 1.55626845 | -3.067 |
| Beta-hexosaminidase | M0T2F6 | 13 | 2.386611 | -3.092 |
| Peroxidase | M0U667 | 5 | 2.34275474 | -3.113 |
| Purple acid phosphatase | M0SF11 | 8 | 2.17377759 | -3.204 |
| Alpha-mannosidase | M0TLF4 | 21 | 2.11246004 | -3.227 |
| Carboxypeptidase | M0SD18 | 8 | 2.09429135 | -3.263 |
| Peroxidase | M0RIL4 | 12 | 2.06259159 | -3.352 |
| Peroxidase | M0SBB3 | 16 | 2.3310055 | -3.355 |
| Lactoylglutathione lyase | M0RNM6 | 8 | 2.61533617 | -3.359 |
| Peroxidase | M0UCQ3 | 3 | 1.89752524 | -3.383 |
| Peroxidase | M0S574 | 13 | 2.50618175 | -3.389 |
| Xyloglucan endotransglucosylase/hydrolase | M0TQQ9 | 5 | 2.34875503 | -3.447 |
| Carboxypeptidase | M0T963 | 5 | 2.66758258 | -3.528 |
| Isochorismatase hydrolase family protein | Q1EP78 | 3 | 2.88370791 | -3.555 |
| Malate dehydrogenase | M0SM75 | 10 | 2.51880923 | -3.582 |
| Vacuolar invertase | H2D4Y7 | 6 | 1.90023567 | -3.586 |
| Carboxypeptidase | M0RG03 | 8 | 2.2668563 | -3.602 |
| Pectin acetylesterase | M0SQ07 | 5 | 2.23937891 | -3.669 |
| Peroxidase | M0S4I5 | 9 | 3.93937839 | -3.692 |
| Pectin acetylesterase | M0SQY8 | 3 | 2.07292216 | -3.705 |
| Lectin | Q8L5H4 | 4 | 2.78840268 | -3.711 |
| Carboxypeptidase | M0S4Z1 | 8 | 2.48383051 | -3.823 |
| Ferredoxin | M0SUM3 | 2 | 2.30331909 | -3.979 |
| Peroxidase | M0TG26 | 4 | 1.90576652 | -3.999 |
| Superoxide dismutase [Cu-Zn] | L7S293 | 4 | 2.605215 | -4.094 |
| Lactoylglutathione lyase OS | M0U4F0 | 5 | 1.81035225 | -4.261 |
| Peroxidase | M0RR04 | 3 | 1.90697123 | -4.338 |
| Ferredoxin--NADP reductase, chloroplastic | M0SSU7 | 4 | 2.72087179 | -4.590 |
| Beta-galactosidase | M0SQP6 | 10 | 2.8996371 | -4.640 |
| Peroxidase | M0T4F6 | 6 | 2.85856565 | -4.980 |
| Purple acid phosphatase | M0TV30 | 4 | 2.73632006 | -5.625 |

Supplemental Table 2. Protein changes of root treated with PEG+SNP against PEG.

| Protein names | Protein IDs | # Unique peptides | Q-Value | Relative abundance  (PEG+SNP/PEG) |
| --- | --- | --- | --- | --- |
| HABP4_PAI-RBP1 domain-containing protein | M0TAE5 | 3 | 1.90107551 | 3.613 |
| DUF2012 domain-containing protein | M0RYK2 | 3 | 1.86566171 | 3.606 |
| Annexin | M0TR77 | 11 | 1.986535317 | 3.069 |
| PHB domain-containing protein | M0RSC6 | 3 | 2.277904486 | 2.889 |
| Histone H4 | M0RV37 | 4 | 1.988738851 | 2.876 |
| Uncharacterized protein | M0RFZ2 | 4 | 2.038918869 | 2.810 |
| Uncharacterized protein | M0TJ00 | 2 | 1.968145117 | 2.724 |
| Importin subunit alpha | M0SHD1 | 9 | 1.956744879 | 2.432 |
| Uncharacterized protein | M0RYU8 | 3 | 2.186776125 | 2.432 |
| AAA domain-containing protein | M0TJN2 | 8 | 3.368825526 | 2.392 |
| PHB domain-containing protein | M0TNP2 | 3 | 2.85890574 | 2.365 |
| Plug_translocon domain-containing protein | M0TGB2 | 3 | 2.366918898 | 2.364 |
| Uncharacterized protein | M0U5C7 | 8 | 2.157366651 | 2.351 |
| Uncharacterized protein | M0TDJ6 | 4 | 2.167977588 | 2.350 |
| Ubiquitinyl hydrolase 1 | M0TW49 | 4 | 1.985706459 | 2.300 |
| Uncharacterized protein | M0U560 | 5 | 2.22727684 | 2.123 |
| Pyruvate kinase | M0TKF8 | 8 | 2.22578535 | 2.108 |
| Ribosomal protein L4 (Fragment) | E9P1K4 | 2 | 3.210177796 | 2.078 |
| Glutamine synthetase | M0U5F8 | 3 | 3.038452736 | 1.905 |
| Uncharacterized protein | M0TYE0 | 3 | 2.181930833 | 1.890 |
| Histone H2B | M0TCC5 | 2 | 2.225042824 | 1.749 |
| Probable bifunctional methylthioribulose-1-phosphate dehydratase/enolase-phosphatase E1 | M0TAQ9 | 6 | 2.682599127 | 1.744 |
| FMN hydroxy acid dehydrogenase domain-containing protein | M0SB12 | 4 | 3.569130826 | 1.710 |
| Aconitase_C domain-containing protein | M0RVV3 | 4 | 2.427526696 | 1.687 |
| Uncharacterized protein | M0TAU3 | 6 | 2.191660276 | 1.589 |
| Malic enzyme | M0RVS9 | 10 | 3.291668301 | 1.536 |
| Ribosomal protein L19 | M0U1Z1 | 2 | 2.407204244 | 1.440 |
| Uncharacterized protein | M0RH07 | 2 | 4.125186694 | 1.402 |
| DHQ_synthase domain-containing protein | M0TIG7 | 7 | 2.506645964 | 1.292 |
| Plasma membrane ATPase | M0RYP4 | 10 | 2.222043778 | 1.280 |
| Fructose-bisphosphate aldolase | M0T5K0 | 5 | 3.305285847 | 1.268 |
| Uncharacterized protein | M0S5F4 | 3 | 3.306483486 | 1.022 |
| Glycine cleavage system H protein | M0SH72 | 2 | 2.544240387 | -1.068 |
| Uncharacterized protein | M0RQQ7 | 5 | 2.40675706 | -1.087 |
| Uncharacterized protein | M0TBK3 | 3 | 2.363906677 | -1.127 |
| Uncharacterized protein | M0T962 | 3 | 2.447049721 | -1.129 |
| Peroxidase | M0S889 | 18 | 2.984294353 | -1.323 |
| FMN_red domain-containing protein | M0SCY4 | 3 | 3.979755453 | -1.376 |
| Uncharacterized protein | M0RUI7 | 5 | 2.775094809 | -1.386 |
| AMP_N domain-containing protein | M0TG16 | 4 | 2.786586301 | -1.458 |
| Uncharacterized protein | M0TQH8 | 6 | 2.582101352 | -1.559 |
| Uncharacterized protein | M0TYD4 | 6 | 2.833579955 | -1.737 |
| Biotin carboxyl carrier protein of acetyl-CoA carboxylase | M0TKZ6 | 3 | 2.231243316 | -1.816 |
| Uncharacterized protein | M0S315 | 2 | 2.470217508 | -1.926 |
| Uncharacterized protein | M0U764 | 4 | 2.490326018 | -1.939 |
| Uncharacterized protein | M0TQE1 | 5 | 2.540888818 | -2.099 |
| FAS1 domain-containing protein | M0TT02 | 5 | 2.52971895 | -2.316 |
| Putative chitinase | Q8VXF0 | 5 | 2.362886219 | -2.396 |
| Xyloglucan endotransglucosylase/hydrolase | M0TQQ9 | 5 | 2.211901069 | -2.627 |
| Uncharacterized protein | M0RVK0 | 2 | 3.636616624 | -4.215 |
